# Effects of Sugammadex and Neostigmine on Post-operative Nausea and Vomiting in ENT Surgery

**DOI:** 10.3389/fmed.2022.905131

**Published:** 2022-05-20

**Authors:** Nik Izyan Syaizana Nik Mat, Chih Nie Yeoh, Muhammad Maaya, Jaafar Md Zain, Joanna Su Min Ooi

**Affiliations:** ^1^Department of Anesthesiology and Intensive Care, Hospital Duchess of Kent, Sandakan, Malaysia; ^2^Department of Anesthesiology & Intensive Care, Universiti Kebangsaan Malaysia Medical Center, Kuala Lumpur, Malaysia

**Keywords:** sugammadex, neostigmine, postoperative nausea and vomiting, reversal of neuromuscular block, ENT surgery

## Abstract

We aim to compare the effects of sugammadex on postoperative nausea and vomiting (PONV) with those of neostigmine–atropine mixture. A total of 136 American Society of Anesthesiology (ASA) I or II patients, aged 18 to 65 years who underwent ear, nose, and throat (ENT) surgery under general anesthesia, were recruited in this prospective, randomized, double-blind study to receive either sugammadex 2 mg/kg or neostigmine 2.5 mg with atropine 1 mg for reversal of neuromuscular blockade. PONV scores and the need for the rescue of anti-emetic were assessed upon arrival in the post-anesthesia recovery unit and at 1-, 6-, 12-, and 24-h post-reversal. The incidence of PONV was significantly lower in patients who received sugammadex (3%) compared to patients who received neostigmine–atropine mixture (20%) at 6 h postoperative (*p* = 0.013). The incidence of PONV was comparable at other time intervals. None of the sugammadex recipients require rescue antiemetic whereas two patients from the neostigmine–atropine group required rescue antiemetic at 1 and 6 h post-reversal, respectively. The need for the rescue antiemetic was not statistically significant. We concluded that reversal of neuromuscular blockade with sugammadex showed lower incidence of PONV compared to neostigmine–atropine combination in the first 6 h post-reversal.

## Introduction

Postoperative nausea and vomiting (PONV) are one of the most unpleasant experience for patients undergoing surgery under general anesthesia and remains a significant problem in modern anesthetic practice because of the adverse consequences such as delayed recovery, unexpected hospital admission, delayed return to work of ambulatory patients, pulmonary aspiration, wound dehiscence, and dehydration (1).

General incidence of PONV reported is in the range of 20–30% but can increase up to 80% in high-risk patients (2). Ear, nose, and throat (ENT) surgeries have a high incidence of postoperative emesis when no prophylaxis is given and the occurrence of nausea or vomiting postoperatively can worsen patients' condition and hence delay recovery and discharge from the hospital (3).

Neostigmine is an anticholinesterase inhibitor used to antagonize muscle paralysis caused by non-depolarizing muscle relaxants through the formation of carbamylated enzyme complex causing increase in the concentration of acetylcholine at the neuromuscular junction (4). It is known to cause bradycardia, increase gastrointestinal motility, and increase gastric secretions (5). Neostigmine is postulated to increase the risk of PONV by provoking gastric spasms, lowering barrier pressure and heighten afferent input to central vomiting center (4). There are several types of receptor for emetogenic neurotransmitters such as dopamine (D_2_) receptors, histaminic (H_1_) receptors, 5-hydroxytryptamine_3_ (serotonin) receptors, and muscarinic cholinergic receptors (6). On this theoretical basis, cholinesterase inhibitor (neostigmine in particular) has been associated with increased PONV (7). Previous study has shown the neostigmine dose of up to 2.5 mg or more increased the risk for PONV (8). However, a meta-analysis of 10 clinical studies involving 933 patients by Cheng et al. demonstrated inconclusive evidence that neostigmine increased nausea or vomiting when given with atropine or glycopyrrolate (9).

Sugammadex is a selective gamma-cyclodextrin drug that terminates the action of muscle paralysis by encapsulating aminosteroid non-depolarizing muscle relaxant (10). It is a fast-onset drug without the muscarinic side effects of neostigmine (11). The well-known side effects of sugammadex were nausea and vomiting but these side effects had been shown to be well-tolerated in adult patients (12). A meta-analysis involving 17 randomized clinical trials that recruited 1,553 patients were unable to conclusively confirm any evidence for the differences in PONV effects between sugammadex and neostigmine (13). Due to these findings, we conducted this study with the aim of comparing the PONV effects when neuromuscular blockade was antagonized with sugammadex compared to neostigmine–atropine combination after ENT surgery.

## Materials and Methods

This was a prospective, double-blinded, randomized clinical study conducted at Universiti Kebangsaan Malaysia Medical Centre (UKMMC) between November 2019 to November 2020. This study was approved by the Research Committee of Department of Anesthesiology and Intensive Care, UKMMC as well as the Medical Research & Ethics Committee, UKMMC (JEP 2019-542).

### Patients

A total of 136 patients aged between 18 and 65 years, American Society of Anesthesiologists (ASA) physical status I or II scheduled for elective ENT surgery, were recruited in the study. Patients who were obese (body mass index >30 kg/m^2^), pregnant, had history of PONV, Apfel score (2) more than 2, impaired renal function (creatinine clearance <30 ml/min), required postoperative ventilation, and allergic to any drugs used in this study were excluded. Patients who underwent middle ear surgery requiring nerve monitoring were also excluded from the study.

### Recruitment and Randomization

During preoperative assessment, patients eligible for the study were assessed for risk of PONV using Apfel score. Patients were then randomized using computer generated sequence to either received neostigmine (Group N) or sugammadex (Group S). Patients were not given sedative premedication prior to their surgery. About 1 g of oral paracetamol was given to the patients prior to operating theater call.

### Methodology

In the operating room, all patients were monitored with standard 3-lead electrocardiogram (ECG), peripheral oxygen saturation (SpO2), and non-invasive blood pressure (NIBP). Baseline pulse rate, systolic blood pressure, diastolic blood pressure, and peripheral oxygen saturation were recorded. Patients received crystalloid infusion of normal saline 0.9% or Hartmann solutions throughout the surgery to replace the loss from dehydration. All patients given general anesthesia were preoxygenated for 3–5 min, followed by administration of intravenous (IV) fentanyl 2 mcg/kg and propofol 2 mg/kg, and paralyzed with rocuronium 0.9 mg/kg before orotracheal intubation. Anesthesia was maintained with sevoflurane to achieve a minimum alveolar concentration of 1.0–1.2 in oxygen and air in a 1:1 ratio.

All patients received prophylactic antiemetics, IV dexamethasone 8 mg on induction of general anesthesia, and IV granisetron 1 mg at the end of surgery. Intravenous parecoxib 40 mg was given 30 min before the end of the procedure as a part of multimodal analgesia management. Additional analgesia of morphine up to 0.1 mg/kg used intraoperatively was recorded. At the end of procedure, volatile agent was terminated, and the patients were ventilated with 100% oxygen. Reversal agent was administered depending on which group the patients have been randomized to receive. Patients in Group N received neostigmine 2.5 mg in combination with atropine 1 mg and patients in Group S received sugammadex 2 mg/kg at the end of surgery. Patients were extubated after suctioning of oropharyngeal secretions and transferred to the postanesthesia recovery unit.

Postoperative nausea and vomiting were assessed by an investigator not involved in the intraoperative care. The incidence of PONV and the need for rescue antiemetics were evaluated for 24 h after surgical procedure. In the postanesthesia recovery unit, nausea and vomiting were assessed using a 4-point verbal descriptive scale as described in previous studies: 0 = not nauseated, 1 = nauseated, not vomiting, 2 = nauseated, one to two episodes of vomiting, 3 = nauseated, more than two episodes of vomiting during the observation period (13). Patients who had vomiting of three or more episodes (PONV score of 3) were given IV metoclopramide 10 mg as rescue antiemetic. Patients were ensured to have stable hemodynamic and adequate pain control prior to discharge to general ward. In the ward, PONV was monitored at 6-, 12-, and 24-h post-reversal with test drugs. Any antiemetic drug given within the first 24 h was recorded. Time to first oral intake that was defined as the time patients started taking food or fluids after the surgery was also recorded.

### Statistical Analysis

In the previous study by Yagan et al. (14), the incidence of PONV was reported as 27% with neostigmine and 7% with sugammadex. Using Schlesselman formula, 62 patients in each group would be required to detect 20% change with 80% power and 5% significance (α = 0.05, β=0.80). We recruited 136 patients in this study to allow for 10% dropouts. Data collected in the study were analyzed using SPSS for MAC version 27.0 (IBM Corp, Armonk, NY, USA). Descriptive statistics were presented as mean ± standard deviation for continuous variable and as number and percentage for nominal variables. Independent sample *t*-test was used for age, BMI, opioid consumption, and duration of surgery. Categorical data such as gender, ASA physical status, and rate of PONV were tested using chi-square or Fisher's exact test. A *p*-value < 0.05 was considered as statistically significant.

## Results

A total of 136 patients were recruited with no dropouts. The demographic data shown in Table 1 were comparable in both groups. There was no statistical significance seen between both groups in terms of PONV risk scores, type of surgery, duration of surgery as well as total morphine consumption intraoperatively.

**Table 1:** Patients and clinical characteristics. Data presented as mean ± SD or number (percentage).

	**Group N (*n* = 68)**	**Group S (*n* = 68)**	***p*-value**
Age (year)	42.9 ± 13.6	39.8 ± 14.3	0.198
BMI (kg m^−2^)	25.9 ± 2.9	25.0 ± 2.6	0.066
**Gender**			0.732
Male	33 (49%)	35 (51%)	
Female	35 (51%)	33(49%)	
**ASA**			0.863
I	37 (54%)	39 (57%)	
II	31 (46%)	29 (43%)	
**Apfel score**			0.678
0	8 (12%)	5 (7%)	
1	27 (40%)	29 (43%)	
2	33 (48%)	34 (50%)	
**Type of surgery**			0.543
Ear	10 (15%)	6 (9%)	
Nose	38 (56%)	39 (57%)	
Pharyngeal	20 (29%)	23 (34%)	
Duration of surgery (min)	137.9 ± 70.3	129.1 ± 62.6	0.441
Morphine consumption (mg)	3.29 ± 1.50	3.62 ± 1.30	0.180

*BMI, body mass index; ASA, American Society of Anesthesiologists*.

Table 2 shows the incidence and severity of PONV on arrival to postanesthesia recovery unit and at 1-, 6-, 12-, and 24-h post-reversal. The incidence and severity of PONV were statistically significant only at 6 h post-reversal (*p* = 0.013). Rescue antiemetic was only required in the neostigmine group at 1- and 6-h post-reversal. The need for rescue PONV was not statistically significant. All patients that scored at least one episode of vomiting in the neostigmine group received neostigmine dose of <45 μg/kg and had received similar amount of morphine intraoperatively (0.05 mg/kg). There was no vomiting noted in either group from 12 h post-reversal onward. None of the patients had PONV by 24 h post-reversal. Time to first oral intake was comparable between both groups. All patients had minimal pain in the first 24 h post-reversal.

**Table 2:** Incidence and severity of PONV in groups. Data presented as number (percentage).

	**Group N (*n* = 68)**	**Group S (*n* = 68)**	***p*-value**
**On arrival to recovery area**			0.381
PONV SCORE 0	35 (52%)	41 (60%)	
1	28 (41%)	25 (37%)	
2	5 (7%)	2 (3%)	
3	0 (0%)	0 (0%)	
**1 h post-reversal**			0.158
PONV SCORE 0	35 (52%)	38 (56%)	
1	26 (38%)	28 (41%)	
2	5 (7%)	2 (3%)	
3	2 (3%)	0 (0%)	
**6 h post-reversal**			0.013^*^
PONV SCORE 0	54 (80%)	66 (97%)	
1	8 (11%)	2 (3%)	
2	4 (6%)	0 (0%)	
3	2(3%)	0 (0%)	
**12 h post-reversal**			0.500
PONV SCORE 0	66 (97%)	67 (99%)	
1	2 (3%)	1 (1%)	
2	0 (0%)	0 (0%)	
3	0 (0%)	0 (0%)	
**24 h post-reversal**			1.000
PONV SCORE 0	68 (100%)	68 (100%)	
**Timing of first oral intake**			0.220
Less than 6 h	63 (93%)	66 (97%)	
More than 6 h	5 (7%)	2 (3%)	

*0 = no nausea or vomiting, 1 = nauseated, no vomiting 2 = nauseated, one or two episodes of vomiting, 3 = nauseated, more than two episodes of vomiting*.

* *p < 0.05 is statistically significant*.

## Discussion

Nausea and vomiting occur more commonly following middle ear or nasal surgery, and least frequently after pharyngeal surgery (15). The incidence of PONV is reported as 62–80% after middle ear surgery and 34–65% after nasal surgery without prophylactic antiemetic medication (16). The higher incidence of PONV in ENT surgery was likely due to the sensory stimulation of the ophthalmic and maxillary divisions of trigeminal nerve in the nose, vagal stimulation from head and neck region, and stimulation of afferent fibers of the vestibular apparatus (15).

Despite middle ear surgery being more emetogenic, our study did not report any severe PONV in either group. Patients who reported severe PONV and required rescue antiemetic at first hour post-reversal were both women in their 30s and underwent nasal (trans-sphenoidal surgery for cerebrospinal fluid leak) and pharyngeal (tonsillectomy) surgery, respectively. Another two patients who needed rescue antiemetic within 6 h post-reversal were in their 30s, one of whom was a woman. Both of them had nasal (septoturbinoplasty) and pharyngeal (tonsillectomy) surgery, respectively as well. We found that all 4 patients had received pharyngeal throat pack insertion during the procedures, and we postulated that the pharyngeal packing may have caused pharyngeal mucosal trauma leading to discomfort and exacerbate PONV (16).

In our study, the average incidence of PONV between 0 and 1 h postoperative was 48% for neostigmine and 40–44% for sugammadex recipients which was comparable between both groups. The rate of PONV in both groups showed similar pattern of decline over time with none of the patients having PONV by 24 h post-reversal. The higher PONV rate for both groups in the early postoperative period in our study was likely attributed to the effects of volatile anesthesia. In a randomized controlled trial by Apfel et al. (17) it was shown that volatile agent was pro-emetogenic and was considered the primary cause of early PONV (0–2 h) with no impact on delayed PONV (2–24 h). As ENT surgery is an emetogenic procedure, prescribing 2 prophylactic antiemetics in our study to mitigate the incidence of PONV have likely reduced the rate of delayed PONV seen in our study (18). Using a combination of dexamethasone and serotonin antagonist, the ability to reduce PONV has been shown to be greater than a single antiemetic agent since these antiemetics act at different receptors (18, 19). Studies have shown that dexamethasone is effective against late PONV (20). In a study done by Rajeeva et al. (21) it was shown that delayed vomiting was better controlled, and nausea score was lesser with combination of ondansetron 4 mg and dexamethasone 8 mg in female patients who underwent laparoscopic gynecology surgery.

In our study, sugammadex showed significantly less incidence of PONV (3%) compared to neostigmine (20%) at 6 h post-reversal. Theoretically, the short duration of action of neostigmine should not give rise to PONV beyond the first hour postreversal. However, cholinesterase inhibitors may decrease esophageal sphincter pressure and increase the secretion of stomach fluid and intestinal movement (7). Unlike what was found in our study, Yagan et al. (14) demonstrated that sugammadex 2 mg/kg showed significantly lower incidence of PONV (8%) compared to neostigmine 50 μg /kg with atropine in the first hour post-operative and less antiemetic used in 24 h of monitoring in a mixed surgical population. The higher incidence for PONV between 0 and 6 h in both of our groups compared to Yagan et al. (14) could be due to the longer duration of surgery in our study averaging 130 min compared to 50 min by Yagan et al. (14). A total of one patient in our study who had severe PONV from 1 h until 6 h post-reversal was found to have a longer duration of nasal surgery (4.5 h compared to 2 h in average for other patients). Apfel et al. (17) had also demonstrated a strong dose–response relationship between duration and use of volatile anesthesia which is pro-emetogenic. It has been established that an increase in surgery duration may increase the incidence of PONV whereby each 30-min increase in duration increases PONV risk by 60%, so that a baseline risk of 10% is increased to 16% after 30 min (22). Tas Tuna et al. (23) reported no significant difference in the incidence of PONV at all time intervals between patients receiving neostigmine 40 μg /kg (with atropine) vs. patients receiving sugammadex 2 mg/kg undergoing laparoscopic cholecystectomy (24). In their study, none of their patients received antiemetic prophylaxis. Similarly, Paech et al. (23)also found no significant difference in PONV between sugammadex 2 mg/kg and neostigmine 40 μg/kg in patients undergoing laparoscopic gynecological procedure. In their study, their patients only received one prophylactic antiemetic (dexamethasone 4 mg) and ondansetron was not given routinely. Instead of atropine, glycopyrrolate was used in combination with neostigmine as reversal agent. In a study by Chhibber et al. (25), neostigmine with atropine was found to be associated with less incidence of PONV compared to neostigmine with glycopyrrolate due to the central anticholinergic action of atropine on antiemesis effect. Similarly, Cheng et al. also found that atropine was associated with a statistically significant decreased risk for PONV compared to glycopyrrolate (9).

Conflicting findings have been reported by several clinical studies regarding the dose of neostigmine used as reversal agent and its relationship with PONV. Koyuncu et al. (5) compared the effects of neostigmine 70 μg/kg and sugammadex 2 mg/kg on PONV in 100 patients undergoing extremity surgery. In their study, patients were not prescribed intraoperative antiemetic, but the author demonstrated that PONV scores were lower only upon arrival in post-anesthesia care area in patients who received sugammadex. PONV was observed in 60% of patients assigned to sugammadex compared to 58% of those that received neostigmine during the initial 24 h postoperative. Higher dose of neostigmine compared to conventional dose of 50 μg/kg might be a contributory factor. Some pieces of literature have linked the higher dose of neostigmine to be a causative factor of PONV (26). High dose of neostigmine (>2.5 mg) is associated with increased PONV (8). Løvstad et al. (27) investigated the effects of neostigmine 50 μg /kg to placebo on PONV on patients with laparoscopic gynecology and found significant increase in PONV during the first 6 h postoperative. In our study, neostigmine was given in a standard dose of 2.5 mg (average of 36 μg/kg). Even though the dose of neostigmine received by patients in our study was lower than the dose used in the previous studies, none of our patients reported any residual paralysis postoperatively, and a study by McCourt et al. showed that neuromuscular blockade induced by rocuronium can be safely antagonized using a neostigmine dose as low of 35 μg/kg (28).

Reducing modifiable risk factors can significantly decrease the rate of PONV. However, clinicians rarely consider the risk of PONV when choosing reversal agents because residual paralysis is a more common concern than PONV. In terms of health economics, Parra-Sanchez et al. performed a comprehensive economic analysis of PONV in patients undergoing ambulatory surgery and they reported an incremental hospital expenditure of $75 per patient which was comparable to the cost that patients would be willing to pay to avoid PONV (29). In a cost analysis study done by Hurford et al. (30), sugammadex would only be a cost-saving strategy in comparison with neostigmine only if neostigmine cost exceeding $84 and a very high likelihood of PONV (30). The conclusion from the study is that they do not support the routine use of sugammadex in patients as a strategy to reduce PONV.

Our study is limited by the fact that it was a single-center study in Malaysia. Also, a shorter assessment time interval within the first 6 h postoperative may be needed to ascertain the peak of PONV due to the short duration of action of neostigmine. Another limitation was that we did not measure any objective biochemical parameters of PONV such as C-reactive protein, ketones, and aldehydes.

## Conclusion

Our study showed that there was significantly less PONV at 6 h post-reversal with sugammadex compared to neostigmine–atropine mixture. We do not advocate the routine use of sugammadex as a means of reducing PONV due to the cost. However, sugammadex when used as a reversal agent in selected cases when poor reversal may be a concern confers the added benefit of reducing PONV, and these dual benefits may far outweigh the cost which cannot be reflected in this study.

## Data Availability Statement

The original contributions presented in the study are included in the article/supplementary material, further inquiries can be directed to the corresponding author/s.

## Ethics Statement

The studies involving human participants were reviewed and approved by Medical Research & Ethics Committee, UKMMC. The patients/participants provided their written informed consent to participate in this study.

## Author Contributions

NM and CY made substantial contribution to the conceptualization, design, methodology, resources, and writing of original draft preparation to this study. CY, MM, JZ, and JO contributed substantially to the review, revision, analysis, and interpretation of data to this study. All authors contributed to the revised final manuscript.

## Funding

This research was funded by UKMMC Fundamental Grant (FF-2019-524).

## Conflict of Interest

The authors declare that the research was conducted in the absence of any commercial or financial relationships that could be construed as a potential conflict of interest.

## Publisher's Note

All claims expressed in this article are solely those of the authors and do not necessarily represent those of their affiliated organizations, or those of the publisher, the editors and the reviewers. Any product that may be evaluated in this article, or claim that may be made by its manufacturer, is not guaranteed or endorsed by the publisher.

## References

[1] Shaikh SI Nagarekha D Hegade G Marutheesh M. Postoperative nausea and vomiting: a simple yet complex problem. Anesthesia, Essays Res. (2016) 10:388–96. 10.4103/0259-1162.179310 27746521 PMC5062207

[2] Apfel CC LCC E Koivuranta M Greim C-A Roewer N. A simplified risk score for predicting postoperative nausea and vomiting: conclusions from cross-validations between two centers. J Am Soc Anesthesiol. (1999) 91:693–693. 10.1097/00000542-199909000-00022 10485781

[3] Mishra AR Srivastava U Kumar D Saraswat N Kumar A Payal YS. Nausea and vomiting after ENT surgeries: a comparison between ondansetron, metoclopramide and small dose of propofol. Indian J Otolaryngol Head Neck Surg. (2010) 62:29–31. 10.1007/s12070-010-0012-x 23120676 PMC3450154

[4] Peck T Harris B. Pharmacology for Anaesthesia and Intensive Care. Cambridge; New York, NY: Cambridge University Press (2021). ISBN: 1108710964.

[5] Koyuncu O Turhanoglu S Akkurt CO Karc Karc M Ozkan M Ozer C. Comparison of sugammadex and conventional reversal on postoperative nausea and vomiting: a randomized, blinded trial. J Clin Anesth. (2015) 27:51–6. 10.1016/j.jclinane.2014.08.010 25544263

[6] Sweis I Yegiyants SS Cohen MN. The management of post-operative nausea and vomiting: current thoughts and protocols. Aesthetic Plast Surg. (2013) 37:625–33. 10.1007/s00266-013-0067-7 23494031

[7] Lee O Choi G Kang H Baek C Jung Y Woo Y. Effects of sugammadex vs. pyridostigmine–glycopyrrolate on postrolate on postx vs. C, Jung Y, Woo Yhts and protocols. Acta Anaesthesiologica Scandinavica. (2017) 61:39–45. 10.1111/aas.12813 27696339

[8] Tramer M Fuchs-Buder T. Omitting antagonism of neuromuscular block: effect on postoperative nausea and vomiting and risk of residual paralysis. A systematic review. Br J Anaesthesia. (1999) 82:379–86. 10.1093/bja/82.3.379 10434820

[9] Cheng C-R Sessler DI Apfel CC. Does neostigmine administration produce a clinically important increase in post-operative nausea and vomiting? Anesth Analg. (2005) 101:1349. 10.1213/01.ane.0000180992.76743.c9 16243993 PMC1458621

[10] Bom A Bradley M Cameron K Clark JK Van Egmond J Feilden H. novel concept of reversing neuromuscular block: chemical encapsulation of rocuronium bromide by a cyclodextrina cyclodextri cyclodext *Angewandte Chemie Int Ed*. (2002) 41:265–70. 10.1002/1521-3773(20020118)41:2%3C265::aid-anie265%3E3.0.co;2-q 12491405

[11] Abrishami A Ho J Wong J Yin L Chung F. Sugammadex, a selective reversal medication for preventing postoperative residual neuromuscular blockade. Cochrane Database Sys Rev. (2009) 4:7362. 10.1002/14651858.CD007362.pub2 19821409

[12] McDonagh DL Benedict PE Kovac AL Drover DR Brister NW Morte JB. Efficacy, safety, and pharmacokinetics of sugammadex for the reversal of rocuronium-induced meeting abstracts in elderly patients. J Am Soc Anesthesiol. (2011) 114:318–29. 10.1097/ALN.0b013e3182065c36 21239968

[13] Abad-Gurumeta A Ripollés-Melchor J Casans-Francés R Espinosa A Martínez-Hurtado E Fernández-Pérez C. systematic review of sugammadex vs neostigmine for reversal of neuromuscular blockade. Anaesthesia. (2015) 70:1441–52. 10.1111/anae.13277 26558858

[14] Yagan Ö Taş N Mutlu T Hanci V. Comparison of the effects of sugammadex and neostigmine on postoperative nausea and vomiting. Rev Bras Anestesiol. (2017) 67:147–52. 10.1016/j.bjane.2015.08.003 27692737

[15] Korkut AY Erkalp K Erden V Teker AM Demirel A Gedikli O. Effect of pharyngeal packing during nasal surgery on postoperative nausea and vomiting. Otolaryngol–Head Neck Surg. (2010) 143:831–6. 10.1016/j.otohns.2010.08.030 21109086

[16] Jin HJ Kim S Hwang SH. Can pharyngeal packing prevent postoperative nausea and vomiting in nasal surgery? Laryngoscope. (2019) 129:291–8. 10.1002/lary.27189 30450698

[17] Apfel C Kranke P Katz M Goepfert C Papenfuss T Rauch S. Volatile anaesthetics may be the main cause of early but not delayed postoperative vomiting: a randomized controlled trial of factorial design. Br J Anaesth. (2002) 88:659–68. 10.1093/bja/88.5.659 12067003

[18] Gan TJ. Postoperative nausea and vomiting—can it be eliminated? JAMA. (2002) 287:1233–6. 10.1001/JAMA.287.10.1233 11886298

[19] Habib AS El-Moalem HE Gan TJ. The efficacy of the 5-HT3 receptor antagonists combined with droperidol for PONV prophylaxis is similar to their combination with dexamethasone. A meta-analysis of randomized controlled trials. Canadian J Anesth. (2004) 51:311ofly 10.1007/BF03018234 15064259

[20] Henzi I Walder B Tramer MR. Dexamethasone for the prevention of postoperative nausea and vomiting: a quantitative systematic review. Anesth Analgesia. (2000) 90:186–94. 10.1097/00000539-200001000-00038 10625002

[21] Rajeeva V Bhardwaj N Batra Y Dhaliwal L. Comparison of ondansetron with ondansetron and dexamethasone in prevention of PONV in diagnostic laparoscopy. Canadian J Anesth. (1999) 46:40–4. 10.1007/BF03012512 10078401

[22] Gijsenbergh F Ramael S Houwing N van Iersel T. First human exposure of Org 25969, a novel agent to reverse the action of rocuronium bromide. J Am Soc Anesthesiol. (2005) 103:695–703. 10.1097/00000542-200510000-00007 16192761

[23] Paech M Kaye R Baber C Nathan E. Recovery characteristics of patients receiving either sugammadex or neostigmine and glycopyrrolate for reversal of neuromuscular block: a randomised controlled trial. Anaesthesia. (2018) 73:340–7. 10.1111/anae.14174 29214645

[24] Tas Tuna A Palabiyik O Orhan M Sonbahar T Sayhan H Tomak Y. Does sugammadex administration affect postoperative nausea and vomiting after laparoscopic cholecystectomy: a prospective, double-blind, randomized study. Surg Laparosc Endosc Percutan Tech. (2017) 27:237–40. 10.1097/sle.0000000000000439 28731951

[25] Chhibber AK Lustik SJ Thakur R Francisco DR Fickling KB. Effects of anticholinergics on postoperative vomiting, recovery, and hospital stay in children undergoing tonsillectomy with or without adenoidectomy. J Am Soc Anesthesiol. (1999) 90:697–700. 10.1097/00000542-199903000-00010 10078669

[26] Gan TJ Diemunsch P Habib AS Kovac A Kranke P Meyer TA. Society for Ambulatory A. Consensus guidelines for the management of postoperative nausea and vomiting. Anesth Analg. (2014) 118:85–113. 10.1213/ANE.0000000000000002 24356162

[27] Løvstad R Thagaard K Berner N Raeder J. Neostigmine 50 μg kg– 1 with glycopyrrolate increases postoperative nausea in women after laparoscopic gynaecological surgery. Acta Anaesthesiologica Scandinavica. (2001) 45:495–500. 10.1034/j.1399-6576.2001.045004495.x 11300390

[28] McCourt K Mirakhur R Kerr C. Dosage of neostigmine for reversal of rocuronium block from two levels of spontaneous recovery. Anaesthesia. (1999) 54:651–5. 10.1046/j.1365-2044.1999.00893.x 10417456

[29] Parra-Sanchez I Abdallah R You J Fu AZ Grady M Cummings K. A time-motion economic analysis of postoperative nausea and vomiting in ambulatory surgery. Canadian J Anesth/J canadien d'anesthésie. (2012) 59:366–75. 10.1007/s12630-011-9660-x 22223185

[30] Hurford WE Welge JA Eckman MH. Sugammadex vs. neostigmine for routine reversal of rocuronium block in adult patients: a cost analysis. J Clin Anesth. (2020) 67:110027. 10.1016/j.jclinane.2020.110027 32980763

